# Relationship between adverse drug reactions to antibacterial agents and the *Klebsiella pneumoniae* carbapenemase-producing (KPC) *Klebsiella pneumoniae* outbreak: insight from a pharmacovigilance study

**DOI:** 10.1186/s40360-019-0364-0

**Published:** 2019-11-12

**Authors:** Milo Gatti, Emanuel Raschi, Fabrizio De Ponti

**Affiliations:** Pharmacology Unit, Department of Medical and Surgical Sciences, Alma Mater Studiorum, University of Bologna, Via Irnerio 48, 40126 Bologna, Italy

**Keywords:** Adverse drug reactions, KPC outbreak, Colistin, Meropenem, Tigecycline, Gentamicin, Ceftazidime/avibactam

## Abstract

**Background:**

The management of *Klebsiella pneumoniae* carbapenemase producing (KPC) infections represents a major challenge. Several safety and efficacy concerns are shared by available antibiotics used in KPC infections, leading to the occurrence of serious adverse drug reactions (ADRs), with ceftazidime-avibactam possibly showing a more favourable risk-benefit profile. We investigated the potential impact of resistance on ADR reports in countries with different prevalence of KPC isolates (Italy vs. United Kingdom [UK]), and described safety profile of newer and older antibiotics used in KPC infections.

**Methods:**

Three spontaneous reporting systems (SRSs) with different features (Italy, UK and worldwide FAERS) were used to describe safety profiles of colistin, meropenem, tigecycline, gentamicin and ceftazidime-avibactam in terms of System Organ Class and Preferred Term level. ADRs were plotted with prevalence of KPC isolates in Italy and UK. A comparison between before-after the KPC outbreak period (1999–2008 vs. 2009–2018) of overall and serious ADRs for selected antibiotics in each SRS was performed. Relationship between total and serious number of ADR reports per year and KPC isolates per year after KPC outbreak (2009–2017) was investigated for both Italy and UK.

**Results:**

A total of 16,329 ADR reports were collected in the three SRSs, with meropenem (42.6%) and gentamicin (36.9%) having the highest number of reports. Significant increase in total and serious ADR reports after the KPC outbreak compared to previous 10 years was found for colistin, meropenem and gentamicin (*p* < 0.01). No significant increase in tigecycline ADRs was reported in FAERS and UK database. Unexpected safety signals involving selected antibiotics were not detected. Significant positive relationship between overall and serious ADR reports and KPC isolates per year for both Italy (*p* < 0.01; *p* = 0.005) and UK (*p* = 0.032; *p* = 0.013) was found.

**Conclusion:**

KPC outbreak led to significant increase in ADRs to selected antibiotics, and a close relationship with antimicrobial resistance was found, both in countries with high and low resistance rate. New safety signals were not detected for selected agents. Active surveillance should be maintained to promptly identify unexpected safety issues.

## Background

The emergence and spread of multidrug-resistant (MDR) and extensively drug-resistant (XDR) *Enterobacteriaceae* have become a public health problem [1]. Particularly, carbapenem-resistant *Enterobacteriaceae* (CRE) are a threat to global health as carbapenems are often considered the “last resort” in the management of antibiotic-resistant Gram-negative infections [2]. Rates of CRE continue to increase globally and invasive infections due to CRE are associated with poor outcomes [3–6]. *Klebsiella pneumoniae* (Kp), by producing the plasmid-encoded enzyme *Klebsiella pneumoniae* carbapenemase (KPC), is the most frequent CRE [7].

The first KPC-Kp producing isolate was identified in USA in 1996 [8], followed by rapid local and global spread. The first outbreaks of KPC-Kp outside the USA were reported in Israel, Greece, China and South America [9]. Currently, the epidemiology of KPC-Kp varies geographically. In Europe, KPC outbreak started in 2009 and continuously increased so far. However, antimicrobial resistance in Northern countries is lower than in Southern European countries [10]. Endemic spread of KPC-Kp has been reported in Italy, Greece, Turkey, Portugal, Cyprus and Romania, while only sporadic diffusion has been observed in many other European countries [9, 10].

Treatment of infections caused by KPC-Kp is challenging, with few antimicrobials available characterized by limited evidence in terms of efficacy and safety [11]. The most frequently used active antimicrobials are “second-line” agents, including colistin, tigecycline, gentamicin, and high-dose carbapenems [12]. The new beta-lactam beta-lactamase inhibitor ceftazidime/avibactam may be a potentially useful antimicrobial in the management of KPC infections, as shown in retrospective observational studies [13, 14].

The safety aspects should not be overlooked when high-doses (carbapenems, tigecycline) or agents with narrow therapeutic windows (colistin, gentamicin) are used to target KPC infections, since the potential increase in serious adverse drug reactions (ADRs) may tip the risk/benefit balance. In this setting, pharmacovigilance by means of actively diagnosing and reporting ADRs may be a useful tool not only to detect early post-marketing risks with new drugs, but also to continue monitoring of older agents [15, 16].

Additionally, the analysis of distinct national pharmacovigilance databases may allow to evaluate the potential impact of different KPC-Kp prevalence (namely high vs. low prevalence) on ADRs reports of antibiotics used in management of KPC infections.

However, to the best of our knowledge, there are no studies investigating the potential correlation between KPC outbreak and ADRs reports of active antimicrobials.

This study aims to investigate the relationship between ADR reporting of agents used in management of KPC infections and endemic spread of KPC, comparing data from Italy (high prevalence of KPC-Kp) and UK (low prevalence of KPC-kp), and to describe safety profile of newer therapeutic strategies for KPC infections, namely ceftazidime/avibactam, as compared to older alternative agents.

## Methods

### Study design

The study was conceived as an observational, retrospective analysis of spontaneous reporting systems (SRSs) combined with microbiological data on antibiotic resistance.

We used a descriptive approach based on unsolicited publicly accessible reports submitted to both international and national SRSs to extract pharmacovigilance data, whereas microbiological data were obtained using publicly available reports provided by the European Centre for Disease Prevention and Control (ECDC). This mixed approach combining two different real-world datasets would allow to (a) identify previously unknown safety issues, (b) provide a public health perspective to ADRs and (c) test the potential relationship between safety issues and antimicrobial resistance.

### Data sources

#### Pharmacovigilance data

Three different SRSs (FDA Adverse Event Reporting System [FAERS] Database, AIFA Database and Yellow Card Scheme) were queried in order to retrieve reports of ADRs for newer and older agents used in KPC treatment, namely colistin, meropenem, tigecycline, gentamicin and ceftazidime/avibactam.

Although the consultation of national SRSs may be insufficient to detect rare events with respect to larger international database, especially in the post-marketing monitoring of newer antibiotics, their use allows to compare results among several countries [17]. Furthermore, the use of national databases provides the actual local picture of the risk, closely associated with the real drug consumption, while avoiding a potential “dilution” phenomenon of reporting pattern that may occur in international databases analysis.

The three SRSs differ as regards data availability, catchment area and date of beginning of ADR collection.

The FAERS database collects worldwide adverse events (US and serious non-US reports) spontaneously submitted by drug companies, healthcare professionals and consumers [18], and offers public access to data from 1968 through the recent public dashboard. The Italian pharmacovigilance database is directly managed by AIFA and contains ADRs collected in Italy from 2002 [19]. Finally, the Yellow Card Scheme is the UK system for collecting information on suspected ADRs to medicines and vaccines [20]. It is maintained by the Medicines and Healthcare Products Regulatory Agency (MHRA) and offers public access to raw data starting from 1964.

#### Microbiological data

Since 2009 the European Centre for Disease Prevention and Control (ECDC) provides annual surveillance reports on antimicrobial resistance, including KPC-Kp, for 30 European countries, including all 28 EU Member States in addition to Norway and Iceland. The results presented in ECDC reports are based on antimicrobial resistance data from invasive isolates (blood and cerebrospinal fluid) collected in European microbiology laboratories and reported to European Antimicrobial Resistance Surveillance Network [21]. The inclusion of isolates obtained only from sterile sites allows to evaluate serious infections characterized by undisputable clinical relevance and requiring a given antibiotic treatment. In order to perform a comparison between countries with different prevalence of KPC isolates and to investigate ADR/resistance relationship, we considered data from Italy (high prevalence of KPC-Kp) and UK (low prevalence of KPC-kp). For both countries, the absolute number of KPC isolates and the proportion of these among the total Kp isolates per year were retrieved from 2009 to 2017.

### Data analysis

First, a descriptive analysis including demographic data (age and sex), number of total and serious ADRs, overall number of drug-System Organ Class (SOC) pairs, frequencies of ADRs in terms of SOC and Preferred Term (PT) levels, and frequency of KPC isolates was performed. For every selected agent, serious ADRs were extracted from each SRS in terms of SOC and PT levels, as codified through the standardized Medical Dictionary for Regulatory Activities (MedDRA) terminology. A serious ADR was defined as “An adverse reaction which results in death, is life-threatening, requires in-patient hospitalization, or prolongation of existing hospitalization, results in persistent or significant disability or incapacity, or is a congenital anomaly/birth defect” [22].

Second, a quantitative-qualitative comparison between the absolute number of total and serious ADRs per year for each selected drug reported from 2009 to 2018 (after KPC outbreak) with respect to the previous 10 years (from 1999 to 2008) was performed. Comparisons were calculated for each of the three different SRSs. Because Italian Medicines Agency (AIFA) Database collected ADRs only from 2002, the historical control was performed on the previous 7 years (from 2002 to 2008) instead of 10 years.

Tigecycline was approved by Food and Drug Administration (FDA) in 2005 and by European Medicines Agency (EMA) in 2006, so the comparison of ADRs pre-post KPC outbreak took into account 4 years (from 2005 to 2008) for FAERS, and 3 years (from 2006 to 2008) for AIFA Database and Yellow Card Scheme.

Qualitative evaluation compared ADR frequencies at SOC and PT levels between the different SRSs, in order to describe safety profile of newer and older agents used for KPC management.

Finally, the existence of a possible relationship between the number of KPC-Kp isolates and the total and serious number of ADR reports after KPC outbreak (from 2009 to 2017) was investigated (pharmacovigilance-microbiological approach). Analysis was performed for both Italy and UK, in order to compare European countries with opposite epidemiological situation in terms of microbiological resistance.

Continuous data were expressed as mean ± standard deviation (SD) and the Student’s *t* test was used for comparison. Categorical variables were expressed as count or percentages, and the Chi-square test or the Fisher’s exact test were used as appropriate.

In order to make a reliable comparison, we have normalized data calculating the mean number of reports per year for the different antibiotics in each SRS. In this way, we obtained data expressing an equal amount and directly comparable using the Student’s *t* test for unpaired data. Similar statistical analyses were performed in order to compare the tigecycline data.

The correlation between the absolute number of total and serious ADRs reports per year for selected agents (x-axis) and the absolute number of KPC isolates per year (y-axis) was assessed by a scatter plot for both Italy and UK. A regression line between the KPC and ADRs prevalence was drawn and the Pearson’s *r* value was calculated. A *p* value of < 0.05 was considered significant.

## Results

Overall, 16,329 ADR reports regarding the selected antibiotics were collected from the three SRSs, of which 90.1% were categorized as serious. Meropenem (42.6% of the overall ADR reports) and gentamicin (36.9%) were the agents with the greater number of reports in every SRSs. Most of reports occurred in patients aged 18–64, except for meropenem, gentamicin and tigecycline in AIFA database, where elderly patients (≥65 years) were preponderant. A slightly higher prevalence of reports in male was detected (47.3% vs. 42.3%) (Table 1).

**Table 1:** Demographic data on overall adverse events with colistin, meropenem, tigecycline, gentamicin and ceftazidime/avibactam in FAERS, AIFA and Yellow Card Scheme Databases. In parenthesis, percentage of total is reported

	Colistin			Meropenem			Tigecycline			Gentamicin			Ceftazidime-Avibactam		
	FDA	UK	IT	FDA	UK	IT	FDA	UK	IT	FDA	UK	IT	FDA	UK	IT
ADR features															
Overall reports	783	87	129	6013	445	494	1879	93	135	5342	407	272	213	14	23
Overall serious reports	737 (94.1)	81 (93.1)	52 (40.3)	5840 (97.1)	377 (84.7)	209 (42.3)	1701 (90.5)	85 (91.4)	64 (47.4)	4901 (91.7)	367 (90.2)	95 (34.9)	180 (84.5)	14 (100)	17 (73.9)
Overall Drug-SOC pairs	2564	284	225	22,557	1019	761	5373	216	219	19,495	823	432	510	32	39
Age															
0–17 years	82 (10.5)	21 (24.2)	6 (4.7)	647 (10.7)	59 (13.3)	43 (8.7)	51 (2.7)	4 (4.3)	2 (1.5)	654 (12.3)	41 (10.1)	41 (15.1)	9 (4.2)	5 (35.7)	0 (0)
18–64 years	396 (50.6)	44 (50.6)	62 (48.1)	2596 (43.2)	240 (53.9)	192 (38.9)	726 (38.6)	45 (48.3)	64 (47.4)	2118 (39.6)	191 (46.9)	107 (39.3)	79 (37.1)	3 (21.4)	8 (34.8)
> 65 years	165 (21.1)	11 (12.6)	41 (31.8)	1964 (32.7)	105 (23.6)	254 (51.4)	583 (31.1)	22 (23.7)	68 (50.4)	1645 (30.8)	140 (34.4)	121 (44.5)	45 (21.1)	2 (14.3)	6 (26.1)
Unknown	140 (17.8)	11 (12.6)	20 (15.4)	806 (13.4)	41 (9.2)	5 (1)	519 (27.6)	22 (23.7)	1 (0.7)	925 (17.3)	35 (8.6)	3 (1.1)	80 (37.6)	4 (28.6)	9 (39.1)
Sex															
Male	369 (47.1)	31 (35.6)	62 (48.1)	3014 (50.1)	195 (43.8)	247 (50)	834 (44.4)	44 (47.3)	68 (50.4)	2421 (45.3)	199 (48.9)	128 (47.1)	88 (41.3)	5 (35.7)	14 (60.9)
Female	306 (39.1)	50 (57.5)	64 (49.6)	2433 (40.5)	217 (48.8)	242 (49)	732 (39)	44 (47.3)	66 (48.9)	2353 (44.1)	183 (45)	142 (52.2)	65 (30.5)	8 (57.1)	6 (26.1)
Unknown	108 (13.8)	6 (6.9)	3 (2.3)	566 (9.4)	33 (7.4)	5 (1)	313 (16.6)	5 (5.4)	1 (0.7)	568 (10.6)	25 (6.1)	2 (0.7)	60 (28.2)	1 (7.2)	3 (13)

*FDA* FAERS Database, *UK* Yellow Card Scheme Database, *IT* AIFA Database

The frequencies of toxicities in terms of SOC and PT levels for each agent partially differed among SRSs (Table 2). “General disorders and administration site conditions” ranked first for all agents in FAERS database, ranging from 9% for gentamicin to 22% for ceftazidime/avibactam. “Skin and subcutaneous tissue disorders” was the most frequently reported SOC in Italy for the selected antibiotics (37.4% of overall drug-SOC pairs), from 12.8% for ceftazidime/avibactam to 48.2% for meropenem. In UK, “investigations” was the most frequently found SOC, from 6.3% for gentamicin to 25% for ceftazidime/avibactam.

**Table 2:** Most frequently reported SOCs and PTs for each antibiotic with proven efficacy against CRE in different spontaneous reporting systems

	Faers		Italy		UK	
	SOC	PT	SOC	PT	SOC	PT
Colistin	General 12.8%	Acute kidney injury 4.8%	Respiratory 16.4%	Acute kidney injury 10.7%	Investigation 17.0%	Acute kidney injury 8.5%
	Renal 8.9%	Drug ineffective 3.0%	Skin 15.6%	Oral paraesthesia 5.3%	Renal 14.1%	Blood creatinine increased 3.2%
	Infections 8.9%	Drug resistance 2.4%	Renal 15.1%	Pruritus 4.9%	General 13.4%	Drug interaction 3.2%
	Respiratory 4.9%	Pathogen resistance 1.8%	Nervous 11.6%	Erythema 4.4%	Nervous 9.4%	Blood urea increased 2.8%
	Nervous 4.7%	Multiple organ dysfunction syndrome 1.7%	General 11.1%	Cough 4.0%	Gastrointestinal 9.4%	Off-label use 2.1%
Meropenem	General 9.9%	Drug ineffective 2.3%	Skin 48.2%	Erythema 11.4%	Skin 14.3%	Neutropenia 3.1%
	Infections 8.0%	Pyrexia 1.9%	Blood 10.8%	Rash 8.4%	General 12.9%	Rash 2.3%
	Investigation 5.9%	Drug interaction 1.4%	General 7.6%	Urticaria 6.3%	Investigation 11.7%	Nausea 2.3%
	Skin 5.7%	Sepsis 1.1%	Respiratory 4.5%	Pruritus 5.0%	Blood 9.3%	Anaphylactic reaction 2.1%
	Blood 5.2%	Thrombocytopenia 1.1%	Gastrointestinal 4.1%	Thrombocytopenia 2.8%	Nervous 8.6%	Pyrexia – Liver function test abnormal 1.8%
Tigecycline	General 13.1%	Drug ineffective 4.1%	Gastrointestinal 28.3%	Nausea 7.8%	General 17.2%	Liver function test abnormal 4.6%
	Infections 9.4%	Death 2.2%	Skin 21.0%	Vomiting 6.8%	Gastrointestinal 16.7%	Drug ineffective 4.6%
	Gastrointestinal 7.9%	Nausea 2.2%	Investigation 11.9%	Erythema 5.9%	Investigation 14.3%	Nausea 3.7%
	Investigation 6.8%	Pancreatitis 2.1%	Hepatobiliary 8.7%	Rash 4.6%	Infections 11.8%	Acute pancreatitis 3.2%
	Blood 5.1%	Sepsis 1.7%	Blood 6.4%	Acute pancreatitis 4.1%	Ear 6.4%	Thrombocytopenia – Death 2.8%
Gentamicin	General 9.0%	Acute kidney injury 4.4%	Skin 40.3%	Erythema 10.0%	Renal 13.9%	Acute kidney injury 8.0%
	Renal 7.6%	Pyrexia 2.1%	Renal 11.8%	Urticaria 7.9%	Ear 11.9%	Hypotension 6.3%
	Infections 5.8%	Drug ineffective 1.4%	General 10.4%	Acute kidney injury 6.3%	Vascular 8.9%	Anaphylactic reaction 5.6%
	Skin 5.3%	Renal failure 1.3%	Eye 6.9%	Rash 6.0%	Nervous 8.4%	Ototoxicity 2.2%
	Investigations 4.6%	Dizziness 1.1%	Nervous 3.9%	Pruritus 4.6%	Immune 8.1%	Deafness 2.1%
Ceftazidime/Avibactam	General 22.0%	Death 6.7%	General 28.2%	Thrombocytopenia 7.7%	Investigations 25.0%	Product use issue 18.8%
	Infections 10.4%	Drug ineffective 5.9%	Infections 20.5%	Direct Coombs test positive 7.7%	Infections 18.8%	Hypernatraemia 15.6%
	Injury 8.0%	Off-label use 4.1%	Skin 12.8%	Septic shock 7.7%	Injury 18.8%	Platelet count decreased 6.3%
	Investigations 6.3%	Pathogen resistance 3.7%	Investigations 10.3%	Multiple organ dysfunction syndrome 7.7%	Metabolism 15.6%	ALT increased 6.3%
	Renal 4.9%	Drug resistance 3.5%	Blood 7.7%	Condition aggravated 7.7%	General 12.5%	Pathogen resistance 6.3%

As regards older agents, acute kidney injury was the most common ADR in all SRSs for colistin and gentamicin. Erythema, rash and neutropenia were the prevalent ADRs for meropenem in national databases. In the case of tigecycline, different issues were detected between the Italian scenario and other contexts. While gastrointestinal and skin disorders dominated in Italy, “drug ineffective” and “death” were the most common ADRs reported at PT level in UK and FAERS databases.

As regards newer therapeutic options for KPC management, few reports involving ceftazidime/avibactam were found in the different SRSs (overall 250 of which 84.4% serious). “General disorders and administration site conditions” and “infections and infestations” were the most frequently reported SOCs in all databases. Considering PT level, in FAERS a high proportion of reports potentially indicating inefficacy (“death” and “drug ineffective”) and microbiological resistance (“pathogen resistance” and “drug resistance”) were recorded. Hypernatremia, thrombocytopenia and direct Coombs test positive were the most prevalent ADRs in national databases.

Comparison between the absolute number of total and serious ADRs reports for each agent in the different SRSs before and after the KPC outbreak is shown in Tables 3 and 4. Significant increase in overall number of ADRs reports from 1999 to 2008 to 2009–2018 was observed for each selected antibiotic, with the exception of tigecycline in FAERS and Yellow Card Scheme database. Likewise, a significant increase in serious ADRs from 1999 to 2008 to 2009–2018 was reported for all selected agents, except for tigecycline in UK.

**Table 3:** Overall ADR reports collected from FAERS, AIFA and Yellow Card Scheme Databases in 1999–2018

Antibiotics	1999–2008^a^	2009–2018^d^	*p* value
Colistin			
FAERS (mean per year ± SD)	8.1 ± 9.6	70.2 ± 51.4	0.00402
AIFA (mean per year ± SD)	0.4 ± 0.8	12.6 ± 9.3	0.0025
Yellow Card Scheme (mean per year ± SD)	1.7 ± 3.3	7.0 ± 3.0	0.0015
Gentamicin			
FAERS (mean per year ± SD)	178 ± 70.5	356.2 ± 176.3	0.0119
AIFA (mean per year ± SD)	6.1 ± 4.3	22.9 ± 6.7	< 0.001
Yellow Card Scheme (mean per year ± SD)	11.5 ± 4.5	29.2 ± 16.2	0.00732
Meropenem			
FAERS (mean per year ± SD)	96.5 ± 46.9	504.8 ± 304.9	0.00214
AIFA (mean per year ± SD)	5.6 ± 2.9	45.5 ± 17.7	< 0.001
Yellow Card Scheme (mean per year ± SD)	9.3 ± 4.3	35.2 ± 9.7	< 0.001
Tigecyclin			
FAERS^b^ (mean per year ± SD)	101.3 ± 56.5	147.4 ± 40.6	0.21
AIFA^c^(mean per year ± SD)	0.7 ± 1.2	13.3 ± 6.3	< 0.001
Yellow Card Scheme^c^(mean per year ± SD)	10 ± 5.3	6.3 ± 3.5	0.35
Ceftazidime-Avibactam^d^			
FAERS (mean per year ± SD)	–	53.3 ± 37.9	–
AIFA (mean per year ± SD)	–	7.67 ± 4.5	–
Yellow Card Scheme (mean per year ± SD)	–	7 ± 4.24	–

*SD* Standard deviation

^a^2002–2008 for AIFA Database

^b^2005–2008 for Tigecycline

^c^2006–2008 for Tigecycline

^d^2016–2018 for Ceftazidime-Avibactam

**Table 4:** Serious ADR reports collected in AIFA and Yellow Card Scheme Databases

Antibiotics	1999–2008^a^	2009–2018^c^	*p* value
Colistin			
AIFA (mean per year ± SD)	0.3 ± 0.5	5 ± 3.7	0.00291
Yellow Card Scheme (mean per year ± SD)	1.6 ± 3.3	6.5 ± 2.9	0.00264
Gentamicin			
AIFA (mean per year ± SD)	2.3 ± 2.4	7.9 ± 5.0	0.015
Yellow Card Scheme (mean per year ± SD)	10.7 ± 4.6	26 ± 14.7	0.00977
Meropenem			
AIFA (mean per year ± SD)	2 ± 1.7	19.5 ± 7.3	< 0.0001
Yellow Card Scheme (mean per year ± SD)	7.9 ± 4.5	29.8 ± 8.5	< 0.001
Tigecyclin			
AIFA^b^(mean per year ± SD)	0.3 ± 0.6	6.3 ± 3.0	< 0.001
Yellow Card Scheme^b^(mean per year ± SD)	9.3 ± 4.6	5.7 ± 3.4	0.30
Ceftazidime-Avibactam^c^			
AIFA (mean per year ± SD)	–	5.7 ± 4.0	–
Yellow Card Scheme (mean per year ± SD)	–	4.7 ± 5.0	–

*SD* Standard deviation

^a^2002–2008 for AIFA Database

^b^2006–2008 for Tigecycline

^c^2016–2018 for Ceftazidime-Avibactam

**Figure 1:**
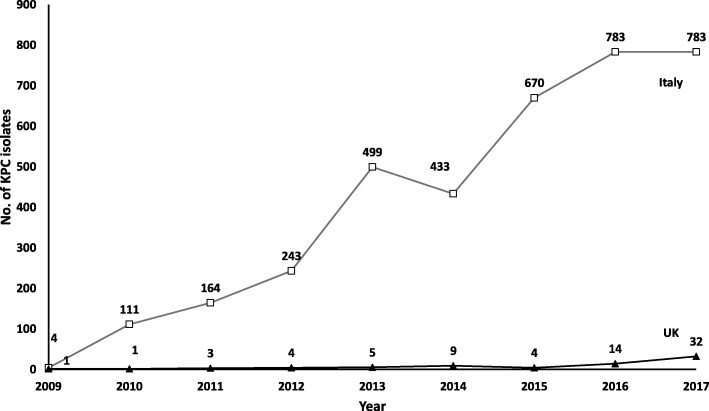
Prevalence of KPC isolates collected in Italy (white squares) and UK (black triangles) from 2009 to 2017. Gradual increase in overall number of isolates was noted for both countries over time

Prevalence of KPC isolates from 2009 to 2017 in Italy and UK is provided in Fig. 1. Over the years, the absolute number of KPC isolates by laboratories cooperating with ECDC continuously increased for both countries.

**Figure 2:**
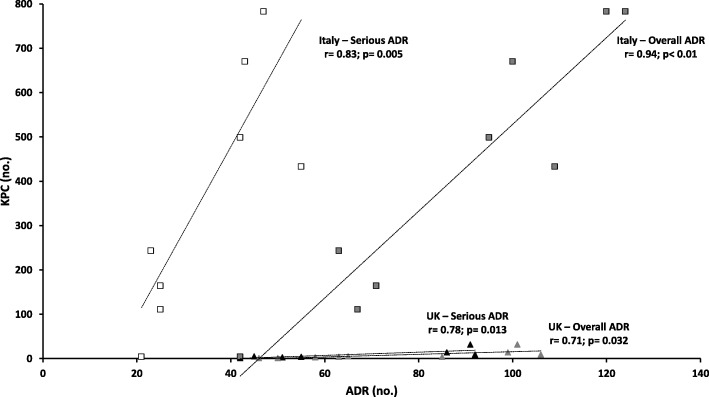
Relationship between carbapenem-resistant Kp isolates per year and overall and serious ADR reports per year concerning agents with proven efficacy against CRE (colistin, meropenem, tigecycline, gentamicin and ceftazidime-avibactam) from 2009 to 2017 (after the KPC outbreak) in Italy (white and grey squares identifying respectively overall and serious ADRs) and UK (black and grey triangles identifying respectively overall and serious ADRs). Significant positive correlations were found for both countries

Significant positive correlation between the two variables were detected in both countries for overall (Italy: r = 0.94; *p* < 0.01; UK: r = 0.71; *p* = 0.032) and serious ADRs (Italy: r = 0.83; *p* = 0.005; UK: r = 0.78; *p* = 0.013) (Fig. 2).

## Discussion

Our study provides a mixed approach in order to investigate the relationship between KPC outbreak and ADRs to antibacterial agents used in the management of this infection, potentially identifying previously unknown safety issues. To our knowledge, this is the first study that combines two different real-world data, examining safety profile of antimicrobial agents used in the treatment of KPC infections and showing positive relationship between the rise of KPC-Kp infections and ADR reports, both overall and serious. Notably, these findings emerged both Italy and UK, despite the wide difference in prevalence of KPC isolates shown by the two countries.

Standard management of KPC infections is based on different combination regimens burdened by limited efficacy and several safety concerns [11, 23], thus it is expected that KPC outbreaks could lead to increase in ADR reports. However, previous studies [5, 6] were only focused on the morbidity and mortality directly associated with KPC infection and pathogen virulence.

Our pharmacovigilance analysis showed worldwide significant increase in ADRs to antimicrobial agents used in KPC infections in the last decade compared to the previous timeframe. Similar findings were found also at national level (Italy and UK). However, significant increase in ADRs to tigecycline after KPC outbreaks was found only in Italy. FDA and EMA safety warnings concerning increased mortality in tigecycline-treated patients versus comparator treatment in pooled clinical trial data [24–29] may have supported clinicians to reduce tigecycline use. On the other hand, in Italy a re-definition of tigecycline treatment in terms of dosage (a double dosage compared to standard management in order to increase the likelihood of achieving optimal target) and indications (targeted use in severe deep-seated infections caused by MDR pathogens or KPC-Kp) was performed [30]. In this context, the widespread use despite safety warnings may explain the rise in ADRs to tigecycline.

It is important to recognize that our pharmacovigilance analysis confirmed safety profiles and known toxicities both of older and newer agents used in KPC infections, in line with findings reported in the literature and relevant Summary of Product Characteristics [31–37]. The lack of unexpected safety signals is reassuring, since clinicians must deal with known adverse events and can use with less concerns newer agents, including Ceftazidime-Avibactam. Although more than 80% of ADRs to ceftazidime-avibactam were classified as serious, mainly involving skin disorders and product use issues, these adverse events are expected and in line with beta-lactam spectrum of toxicities [38].

Although “infections and infestations” represent one of the most frequently reported SOCs for each selected agent in FAERS database, indication bias may have occurred in most reports, causing a distorted association between exposure and outcome. Similarly, “drug ineffective” and “death” are the most frequently found PTs in FAERS. However, in line with recent data [39], it is debated whether this ADRs represent real lack of efficacy or should be interpreted as an infection-related complication in critically ill patients. Although for antibacterial agents used in treatment of KPC infections these ADRs account for 12.2% of serious reports, we found a significant rise in serious ADRs after KPC outbreak for colistin, meropenem and gentamicin also after excluding adverse events classified as “drug ineffective” and “death”.

We acknowledge some limitations of this study, first of all the inability to infer a causal relationship between drug exposure and occurrence of ADR, as well as other sources of bias (under- and over-reporting, missing data) precluding the risk assessment and ranking among drugs. However, pharmacovigilance studies are unreplaceable to investigate safety profiles of medications and identify emerging toxicities for optimizing safety prescribing. Second, the increase in ADRs reports of selected agents in the recent past may be associated with the spread of MDR infections, and not only necessarily related to KPC outbreak. Additionally, ECDC data on absolute number of KPC per year in each European country represent only a fraction of the total amount of infections collected, while the overall number of ADR reports for any drug per year provided by each spontaneous reporting system is available. Consequently, the implementation of an ADR/KPC ratio assessing the number of KPC infections needed for the occurrence of an ADR report was not possible. Finally, in recent years, a progressive and constant rise in overall ADR reports was noted (e.g. in FAERS database a 4-fold increase from 1999 to 2008 to 2009–2018), as a result of raising awareness among patients and healthcare professionals, and implementation of computerized reporting systems easier to use. This could partly explain the largest amount of reports involving antibiotics used in KPC treatment in the last decade. However, for colistin and meropenem a 8.7-fold and 5.3-fold increase in ADR reports after KPC outbreaks was respectively found, also after excluding “drug ineffective” and “death” events. Before release of ceftazidime-avibactam, colistin was the only active antibiotic for most of KPC isolates, and the combination regimen including colistin and high-dose meropenem showed the greater clinical cure rate compared to other antibiotic schedules [11, 12]. Consequently, the increase in ADR reports to colistin and meropenem in the last decade may be closely related to KPC outbreak.

## Conclusions

In conclusion, our approach combining microbiological and pharmacovigilance data may be useful and promising in the assessment of safety issues in cases of bacterial outbreaks, providing comparison among different healthcare settings. KPC outbreak led to significant increase in ADRs to selected antibiotics, and a close relationship with antimicrobial resistance was found, both in countries with high and low resistance rate. New safety signals were not detected for selected agents. Notwithstanding the observed increase in serious ADRs to antimicrobial agents used in KPC infections, the lack of unexpected safety signals for older antibiotics is reassuring. Additionally, no new safety signals emerged with the use of ceftazidime-avibactam. Further pharmacovigilance studies are warranted to continue monitoring of the safety profile of these agents. Considering the expected increasing use of Ceftazidime-Avibactam and the relatively-short time on the market, clinicians should not overlooked the potential occurrence of rare idiosyncratic ADRs, and submit relevant adverse events to regulatory authorities; this will support pharmacovigilance experts in active routine monitoring and assessment of its risk/benefit profile.

## Data Availability

All data collected and used in our study were retrieved from pharmacovigilance database offering public access at the following links: - https://www.fda.gov/drugs/fda-adverse-event-reporting-system-faers/fda-adverse-event-reporting-system-faers-public-dashboard. - http://www.aifa.gov.it/content/online-i-dati-sulle-segnalazioni-di-sospette-reazioni-avverse-registrate-nella-rete-nazional. - https://yellowcard.mhra.gov.uk/iDAP/. - https://ecdc.europa.eu/en/antimicrobial-resistance/surveillance-and-disease-data/report.

## Abbreviations

ADR: Adverse drug reaction
AIFA: Italian Medicines Agency
CRE: Carbapenem-resistant *Enterobacteriaceae*
DDD: Defined daily dose
ECDC: European Centre for Disease Prevention and Control
EMA: European Medicines Agency
FAERS: FDA Adverse Event Reporting System
FDA: Food and Drug Administration
Kp: *Klebsiella pneumoniae*
KPC: *Klebsiella pneumoniae* carbapenemases
MDR: Multidrug-resistant
MedDRA: Medical Dictionary for Regulatory Activities
MHRA: Medicines and Healthcare Products Regulatory Agency
PT: Preferred Term
SD: Standard deviation
SOC: System Organ Class
SPC: Summary of Product Characteristics
SRS: Spontaneous reporting system
UK: United Kingdom
XDR: Extensively drug-resistant
